# Diversity of Omicron sublineages and clinical characteristics in hospitalized patients in the southernmost state of Brazil

**DOI:** 10.1186/s12879-024-09089-3

**Published:** 2024-02-13

**Authors:** Jaqueline Rhoden, Andressa Taíz Hoffmann, Janaína Franciele Stein, Mariana Soares da Silva, Juliana Schons Gularte, Micheli Filippi, Meriane Demoliner, Viviane Girardi, Fernando Rosado Spilki, Juliane Deise Fleck, Caroline Rigotto

**Affiliations:** 1 Laboratório de Microbiologia Molecular, Universidade Feevale, Rodovia ERS-239, n. 2755, Prédio Vermelho, Piso 1, sala 103, Vila Nova, CEP 93525-075, Novo Hamburgo, Rio Grande do Sul Brazil; 2 Santa Casa de Misericórdia de Porto Alegre, Hospital Dom Vicente Scherer, Centro Histórico. Av. Independência, n. 155, CEP 90035-074, Porto Alegre, Rio Grande do Sul Brazil

**Keywords:** SARS-CoV-2, NGS, Sequencing, Vaccination

## Abstract

**Background:**

Omicron has become the dominant variant of severe acute respiratory syndrome coronavirus 2 (SARS-CoV-2) since first reported in November 2021. From the initially detected Wuhan lineage, sublineages BA.2, BA.4, BA.5, BQ, XAG, and XBB have emerged over time and are dominant in many countries. Therefore, the aim is to evaluate which variants are circulating and the clinical characteristics of inpatients infected with the Omicron variant.

**Methods:**

This retrospective cohort study selected hospitalized patients admitted with respiratory symptoms to a hospital in the state of Rio Grande do Sul, Brazil, between June and July 2022. SARS-CoV-2 results were analyzed together with clinical outcomes and vaccination status. A viral genome library was prepared and forwarded to the Illumina MiSeq Platform for sequencing.

**Results:**

In total, 37 genomes were sequenced. Concerning the Omicron sublineages, our study detected: BA.1 (21 K), BA.2 (21 L), BA.4 (22A), BA.5 (22B), BA.2.12.1 (22C), BQ.1 (22E), XBB (22F), and XAG recombinant. Omicron BA.5 (30%), BA.2 (19%), and BQ.1 (19%) were the most frequent sublineages, respectively. In total, 38% of patients present hypertension, and the most common symptoms were coughing (62%). Analyzing the COVID-19 vaccination, 30% of patients were fully vaccinated, 49% had a partial vaccination status, and 21% were unvaccinated (no dose).

**Conclusions:**

BA.5 was the most prevalent sublineage in our study and surpassed the predominance of BA.2, as reported by the national genomic surveillance program. BQ.1 was diagnosed earlier in this study than it was officially reported in the state. Current data have demonstrated that the Omicron variant causes less severe infections, with the high rate of transmissibility and mutational landscape causing the rapid emergence of new sublineages.

**Supplementary Information:**

The online version contains supplementary material available at 10.1186/s12879-024-09089-3.

## Background

On 24 November 2021, the World Health Organization (WHO) reported a new variant of SARS-CoV-2 (severe acute respiratory syndrome coronavirus 2): B.1.1.529, from South Africa. This variant of concern (VOC) was designated Omicron based on the changes in coronavirus disease 2019 (COVID-19) epidemiology, the increase in the number of cases due to the great potential for dissemination, and the risk of reinfection. Previously identified VOCs (Alpha, Beta, Gamma, and Delta) emerged when the vaccination measures for COVID-19 were being established. Omicron appeared at a time when global immunity had been achieved through available vaccines [1, 2].

A large number of new mutations in the spike protein led to the Omicron variant, the most mutated SARS-CoV-2, compared to the first Wuhan lineage described at the beginning of the disease [3]. More than 60 mutations in its genome, 32 of which are in the spike protein’s receptor binding domain (RBD) [3, 4]. Many of them were observed previously in the Alpha (del69–70), Kappa and Iota (T95I), and Kappa and Delta (G142D) variants. Mutations in the Omicron spike region fall in the footprint of the main virus entry receiver, the human angiotensin-converting enzyme 2 (ACE2). Thus, mutations can provide a potential and evolutionary advantage by strengthening the virus ACE2-RBD binding [4, 5].

Initially, in November 2021 the sublineages BA.1, BA.2, and BA.3 were discovered simultaneously and share many common mutations, though each also has unique characteristics. Subsequently, in January and February 2022, BA.4 and BA.5 were defined and presented spike proteins most closely related to BA.2, indicating a common ancestor [5]. BA.4 and BA.5 have identical mutations in the 5′ region (ORF1ab to envelope) yet exhibit divergence in the 3′ region (from M to the 3′ genome end). Therefore, it is related to a recombination event [4, 6]. With the circulation of the Delta variant and Omicron emerging, recombination events occurred, and new sublineages appeared, like XBB and BQ.1 [5, 7, 8].

In summary, the Omicron variant is characterized as a VOC with increased infectivity and an enhanced capacity to evade the immune system, hence a resurgence in cases, hospital admissions, or deaths. In the South of Brazil, as of December 2021, this VOC caused the fifth wave of COVID-19 cases with new infections associated with higher transmissibility. The symptoms of the infection are less dangerous than those of other SARS-CoV-2 strains, but it is more transmissible and less susceptible to vaccines [3, 5, 6].

Following the initiation of vaccination programs around the world, the pandemic scenario has changed. In Brazil, the immunization panel standardized by the Brazil Ministry of Health defined the vaccination number of doses to each patient based on age. However, the spread of Omicron is likely to have important implications such as an increase in the number of cases. In addition, strategies are needed to limit transmission. The main goal of this study was to evaluate the circulation of Omicron lineages and the clinical characteristics of hospitalized patients in a hospital in Rio Grande do Sul (RS), a state in southern Brazil. As the population exhibits a diverse vaccination profile and little is known about how the new sublineages have affected individuals, this study aims to assess the clinical profile of patients, including symptoms and comorbidities, as well as the outcome of the infection. The continued discovery of diverse Omicron lineages can leverage understanding around the evolution and spread of the virus.

## Methods

### Sampling

This study was approved by the Research Ethics Committee at Santa Casa de Misericórdia de Porto Alegre Hospital (file number: 57888422.3.0000.5335 - Ethical Review Presentation Certificate). June and July are the coldest months of winter, and therefore patients admitted to a hospital in the city of Porto Alegre, in the state of Rio Grande do Sul, were selected in 2022. As inclusion criteria for our study, the patient had to be hospitalized and exhibit respiratory symptoms. There was no age limitation. After the selection and to avoid possible bias, the patients were classified in age categories.

Clinical characteristics like symptoms, comorbidities, and clinical outcomes were collected directly from the medical records (Tasy 3.07.1815.133 Software) of each patient and accessed at the hospital. COVID-19 vaccine status was collected through the national immunization program, and the classification was defined according to the immunization panel standardized by health normative from the Brazilian Government, based on age [9]. During the period of study, patients considered fully vaccinated included those aged over 40 years old (with 4 doses), between 12 and 39 years old (with 3 doses), and between 5 and 11 years (with 2 doses). Children under 4 years old were not included in the panel during the period of this study. Considered partially vaccinated patients with a different number of doses than indicated, as described above.

### SARS-CoV-2 detection and genome sequencing

Naso-oropharyngeal swab samples were submitted to nucleic extraction, and infections were confirmed through molecular methods, as follows. Initially, the commercial MagMAX™ CORE Nucleic Acid Purification Kit (Applied biosystems™, Thermo Fisher Scientific, Waltham, MA, USA) was used to perform viral RNA extraction using automated KingFisher™ Duo Prime (Thermo Fisher Scientific™) equipment. For SARS-CoV-2 detection, reverse transcription-quantitative real-time polymerase chain reaction (RT-qPCR) was performed using the Charite Institute (Berlin, Germany) protocols for selecting the envelope (E) gene. Assays were performed with AgPath-ID One-Step RT-PCR Reagents (Thermo Fisher Scientific™).

Viral genome sequencing was carried out with the COVIDSeq Illumina Kit, following the manufacturer’s instructions (San Diego, CA, USA). The viral genome library used was the Illumina MiSeq platform (Foster City, CA, USA), with MiSeq Reagent Kit v3 (600-cycle). The Geneious Prime™ (2023.0.1) suite was used for genome assembly, editing, and mapping the sequences against the reference sequence (NC_045512) available in the EpiCoV database from the Global Initiative on Sharing Avian Influenza Data (GISAID) [10]. PANGO and Nextrain lineage assignments were applied to characterize the consensus sequences.

Sequences generated in this study were aligned with 116 Brazilian SARS-CoV-2 complete genomes and the reference sequence that were retrieved from the GISAID database. Alignment using the reference sequence from Wuhan as the outgroup was performed using Geneious Prime™. The evolutionary history was inferred by using the Maximum Likelihood method and General Time Reversible model [11]. The tree with the highest log likelihood (− 48,488.33) is shown. The percentage of trees in which the associated taxa clustered together is shown above the branches. Initial tree(s) for the heuristic search were obtained automatically by applying Neighbor-Join and BioNJ algorithms to a matrix of pairwise distances estimated using the Maximum Composite Likelihood (MCL) approach (applying 1000 bootstraps), and then selecting the topology with superior log likelihood value. A discrete Gamma distribution was used to model evolutionary rate differences among sites (5 categories (+G, parameter = 1.6111)). The rate variation model allowed for some sites to be evolutionarily invariable ([+I], 48.94% sites). The tree is drawn to scale, with branch lengths measured in the number of substitutions per site. This analysis involved 152 nucleotide sequences. There was a total of 29,921 positions in the final dataset. Evolutionary analyses were conducted in MEGA11 [12].

## Results

From a total of 64 patients positively diagnosed with COVID-19, and following confirmation, samples with cycle threshold (Ct) below 30 were selected, according to laboratory protocol cut-off. As such, 37 genomes were sequenced. Of them, 29 (78%) were from June and 8 (22%) from July. Patients were from different cities in the state of Rio Grande do Sul, female samples corresponded to 21 (57%) and male to 16 (43%), with ages ranging from 11 months to 97 years old, while the average age recorded was 51 years old.

**Fig. 1:**
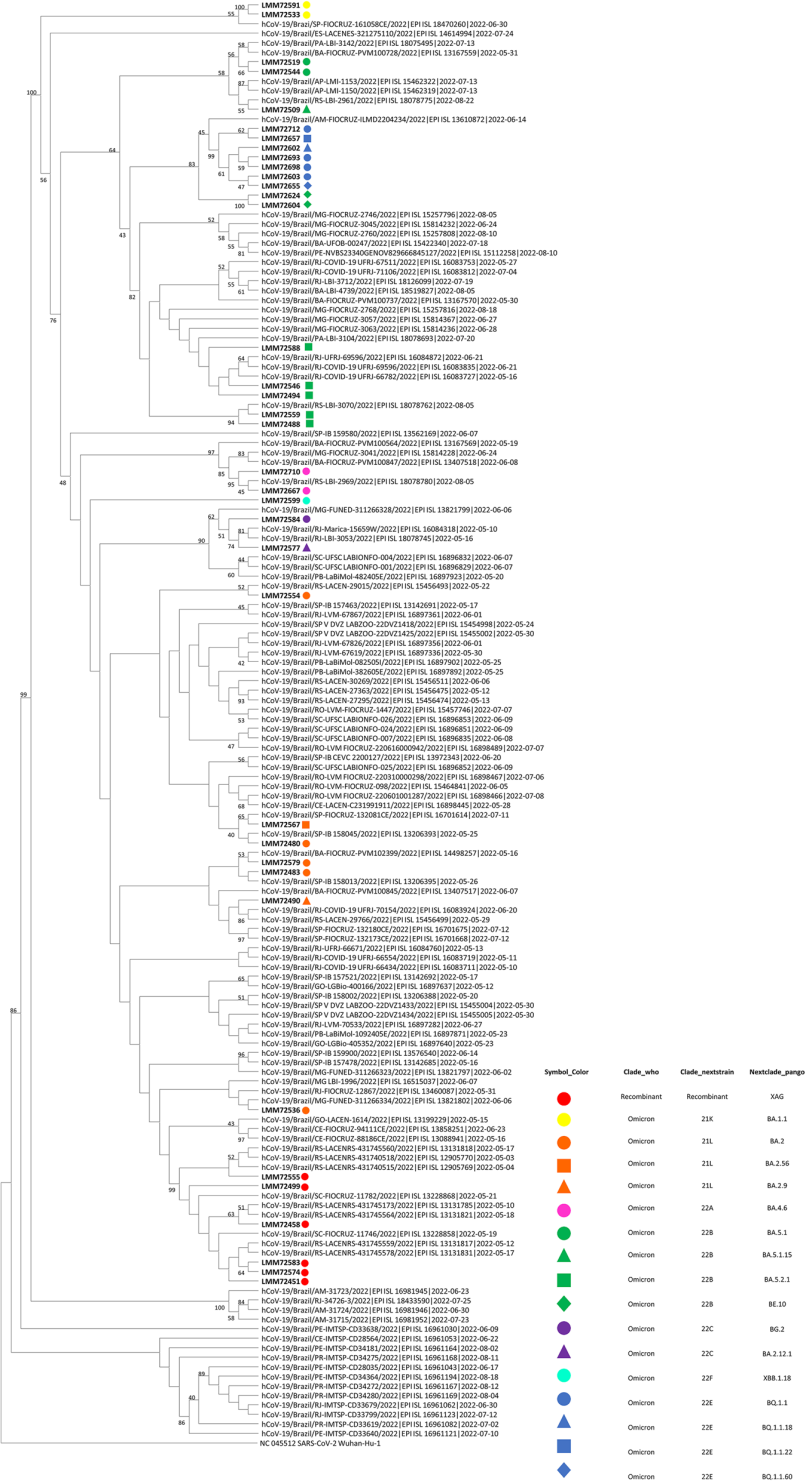
Phylogenetic analysis of complete genomes of SARS-CoV-2 Omicron variant in patients and alignment with other Brazilian SARS-CoV-2 strains. Maximum Likelihood method and General Time Reversible model; Maximum Composite Likelihood applying 1000 bootstraps

**Fig. 2:**
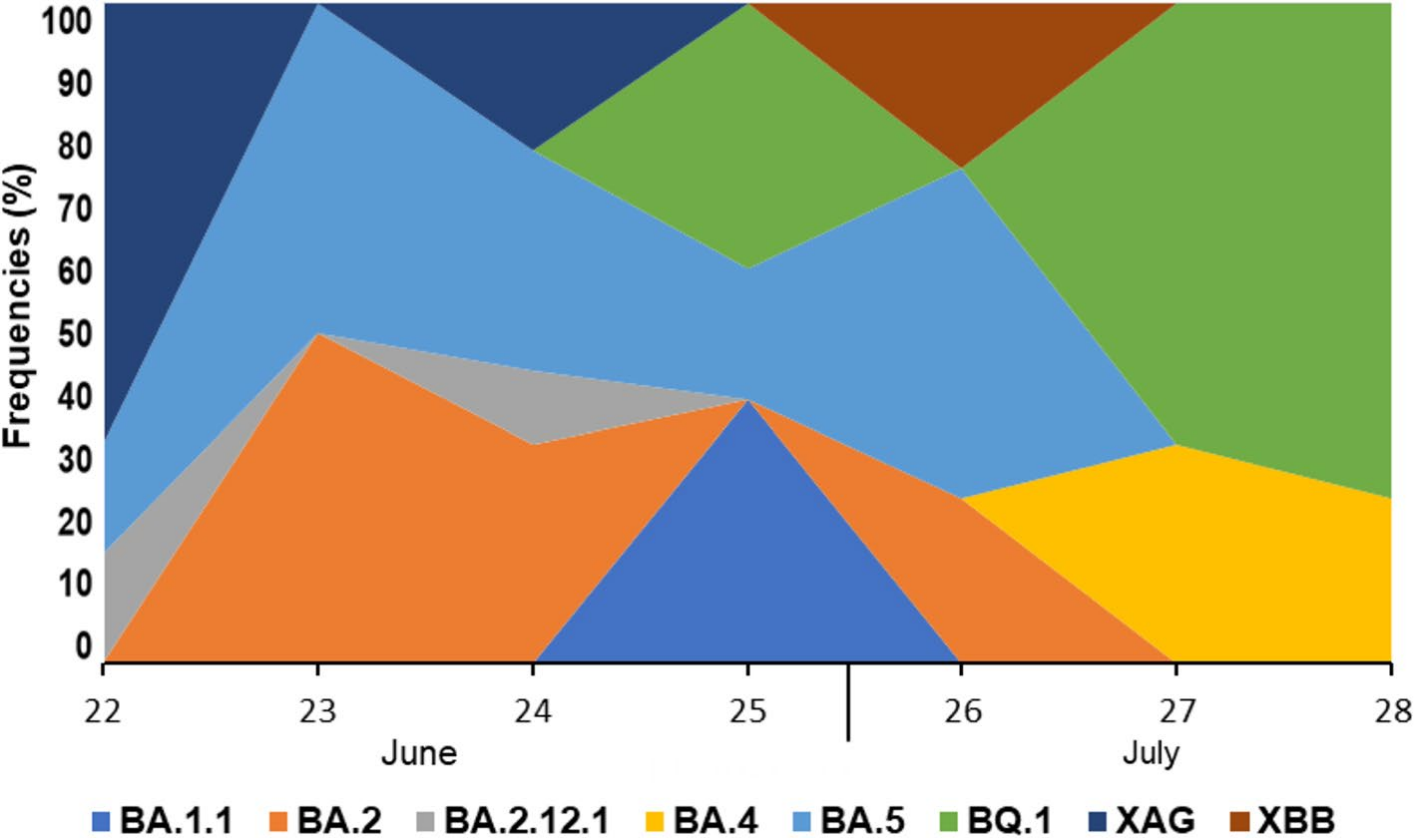
Omicron sublineage distribution by epidemiological weeks

With the genetic characterization, the phylogenetic tree was inferred (Fig. 1). All sequences were uploaded to GISAID, and the accession numbers can be observed in the Supplementary Material 1. Likewise, coverage depth, coverage breadth, and additional information about the sequences are in the Supplementary Material 2. The Omicron variant represented 100% of the frequency of the sequences generated. BA.1 (21 K), BA.2 (21 L), BA.4 (22A), BA.5 (22B), BA.2.12.1 (22C), BQ.1 (22E), XBB (22F), and XAG recombinant sublineages were detected, and the most prevalent sublineages were from the Omicron BA.5 (30%). Subsequently, other frequencies were BQ.1 (19%), BA.2 (19%) and XAG (16%). The representation of sublineage detection distribution is depicted in Fig. 2 across epidemiological weeks, with weeks 22–25 corresponding to June and weeks 26–28 to July.

Analyzing the clinical characteristics, summarized in Table 1, 38% of patients present hypertension, 9% had one kind of malignance and 19% had neurological disease. The most common symptoms were coughing (62%), fever (46%), and headache (40%). Of the total number of patients, there were three deaths, two of whom suffered from lymphoma. The deaths corresponded to a 10-year-old female child, infected with Omicron BA.1.1; a 42-year-old adult man, infected with BA.5; and a 75-year-old female, infected with BA.2. We did not have access to the patient’s cause of death, as it was not described in the medical records.

**Table 1:** Clinical characteristics of patients infected with the SARS-CoV-2 Omicron variant

Characteristics	No. (%)
**Age groups, year**	
Children (0–12)	10 (27%)
Adults (13–59)	8 (22%)
Elderly (> 60)	19 (51%)
Middle age	51 years
**Sex**	
Female	21(57%)
Male	16 (43%)
**Symptoms**	
Cough	23 (62%)
Fever	17 (46%)
Headache	15 (40%)
Coryza	9 (24%)
Vomit	5 (13%)
Sore throat	3 (8%)
**Comorbidities**	
Hypertension	14 (38%)
Malignancies	9 (24%)
Neurological disease	7 (19%)
Cardiovascular disease	6 (16%)
Diabetes	5 (13%)
Obesity	4 (11%)
Chronic obstructive pulmonary disease (COPD)	2 (5%)
**COVID-19 vaccination status**	
Unvaccinated ^a^	8 (21%)
Partially ^b^	18 (49%)
Vaccinated ^c^	11 (30%)
**Clinical outcomes**	
Hospital discharge	34 (92%)
Death	3 (8%)

^a^ Unvaccinated: a person who received no COVID-19 vaccination, according to the immunization panel, ^b^ Partially vaccinated: a person who received a COVID-19 vaccine dose but had not completed the immunization panel, ^c^ Vaccinated: a person who completed a COVID-19 vaccine dose, in accordance with the immunization panel

**Fig. 3:**
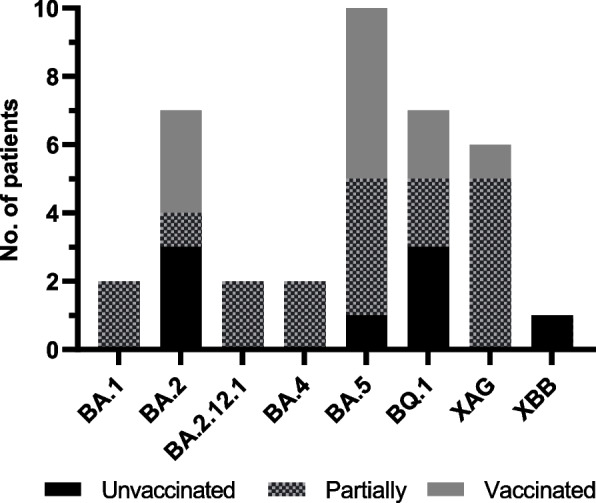
Vaccination status and number of patients according to sublineages detected

During the study, the COVID-19 full vaccination panel varied depending on age. Of the total, 30% of patients were fully vaccinated, 49% had a partial vaccination status, and 21% were unvaccinated (no dose). The relationship between vaccination status and the Omicron sublineage is shown in Fig. 3.

## Discussion

Until July 2022, the southern region of Brazil had the highest incidence of COVID-19 cases in the country. The circulation of the BA.2 sublineage in Brazil represents 61.5% of the genomes sequenced in the overall population, according to data published in the national genomic surveillance program [13, 14]. Omicron BA.2 emerged in Brazil in early January 2022, and according to Silva et al. (2022), the BA.2 sublineage represented 64% of the genomes sequenced in June 2022. In Minas Gerais, another Brazilian state, Queiroz et al. [15] showed BA.1 as the most present sublineage when the number of cases declined and BA.2 became prevalent. Further, proving highly successful at evading the host immune system, BA.4 and BA.5 replaced all the previously emerged sublineages and became dominant globally.

In the state of Rio Grande do Sul, the first cases of BA.5 infections were reported in late May and early June 2022. Monitoring data showed that during the time of the study, BA.2 was the most frequent (75%) sublineage detected in the state [16]. This frequency fails to correspond with the findings in this study, where BA.5 was detected in 30% of samples and BA.2 in 19%. Although, this corroborates with studies in other regions of Brazil and countries where the BA.5 sublineage presented a wave of infections during this time [4, 17, 18]. According to Sousa et al. (2023), BA.5 represents 41.1% of the sequenced genomes in the Maranhão, state in the northeastern region of Brazil.

As of October 2022, BQ.1 has been detected in 65 countries with a prevalence of 6% and represents one of the most recently discovered sublineages. According to Scarpa et al. (2022), the genome has a close relationship with its direct progenitor BA.5 and is typically characterized by amino acid mutations involving important genes. In this study, BQ.1 was detected in seven patients (19%), although the first official related case had been reported in November 2022 by the Rio Grande do Sul State Health Surveillance Center. In other Brazilian states, studies have documented the introduction of this sublineage since November 2022 [18]. The elevated potential to replace BA.5 positioned BQ as a new dominant variant.

With the circulation of different variants in the same place and at the same time, chronic infection and co-infection become possible through viral recombination. A genome recombination event, like circulating lineages combined with the host, is an important evolutionary mechanism for the emergence of pathogens [5, 14]. XAG was characterized by four unique mutations like synonymous mutations and is a recombination of Omicron BA.1.1 and BA.2.23, while recombinant XBB has a large portion of the mutations in the spike protein derived from BA.2 [19].

XAG was first identified in the state in April 2022 and detected in our study in 16% of samples. This result is similar to the findings in other studies and monitoring services [16, 20]. In a study conducted by Silva et al. (2022), an examination of samples collected from various Brazilian states revealed the identification of 252 sequences attributed to the recombinant XAG cluster as of July 2022. The XBB recombinant was detected once (3%), and the first official detection declared by WHO occurred in October 2022. Ao et al. (2023) reported a global prevalence of 1.3% and the spread to 35 countries. The BQ sublineage, even as XBB presented immune evasion through enhanced receptor-binding affinity, suggests a higher reinfection risk and is one advantage over other circulating Omicron sublineages [19].

The infectivity of Omicron is considered higher than the ancestral SARS-CoV-2 variant principally due to BA.4/BA.5, though the severity of illness, hospitalization, and deaths are lower [17]. Analyzing the clinical outcomes found in this study showed no relationship with one specific variant, sex, and age. Among the comorbidities presented by the patients, two had lymphoma. They were also admitted to the intensive care unit, and needed intubation, and the clinical outcome was death. The others were not related to the severity and SARS-CoV-2 infection. These clinical features with lower severity were consistent with further findings in studies. BA.4 and BA.5 are known to have a growth advantage over other variants because of improvements in intrinsic transmissibility or enhanced immune evasion [5, 17, 21]. No relationship between comorbidities, symptoms, and sublineages was found in this study.

COVID-19 vaccination had conferred robust protection against clinical disease involving the BA.1.1 sublineage, right during the first infection peak. The lower incidence of hospitalization and death, and shorter-lasting symptoms are in direct association with the high level of vaccination and previous infections by other variants and so afford some protection [5, 17]. Evaluating the vaccination status of patients who died, the child was partially vaccinated, the adult was fully vaccinated, and the elderly person was unvaccinated. Patients received at least one dose corresponding to 83% (31/37), which attests to the effectiveness of the protective measure.

According to Lewnard et al. (2023), 15.8% of the BA.4/BA.5 cases had not received any vaccine doses, and among BA.2 cases the number was 16.1%. The index in this study corresponds to 8 and 43%, respectively. These findings suggest that BA.4/BA.5 infections reach individuals with greater immune protection against SARS-CoV-2 than BA.2. Studies showed that three or four doses of vaccine do not induce robust neutralization against BA.4/BA.5 since the spike protein mutations confer a capacity of immune evasion. Moreover, XAG and BQ.1 exhibit the greatest evasion against vaccine neutralization and suggest that these new sublineages will replace BA.5 as the dominant variant in circulation [17, 20]. This analysis is limited to a restricted number of samples to conclude the relationship between vaccination status and Omicron infection.

The severity of infections is influenced by variant-specific properties and the intrinsic capacity to transmit and infect another individual with immunity or not, as from vaccination or infection. This study presents different data on the circulation of Omicron sublineages in the southernmost state of Brazil that have been published so far. Our results represent the analysis of hospitalized patients. The analysis of this population yields interesting and debatable results, unlike the general population. It also highlights the detection of BA.5 as the most prevalent sublineage surpassing the predominance of BA.2 reported by the national genomic surveillance program. BQ.1 was also diagnosed before it was officially reported. Surveillance of sublineages circulation helps rapidly identify new emerging lineages, so can develop better prophylactic measures.

### Supplementary Information

**Supplementary Material 1.**

**Supplementary Material 2.**

## Data Availability

All data relevant to the study are included in the article and additional files. The SARS-CoV-2 genomes were deposited in the global initiative on sharing all influenza data (GISAID) database (https://www.gisaid.org/). GISAID Identifier: EPI_SET_240202vg, available at 10.55876/gis8.240202vg.
