# Supplementary material for: Diversity of Omicron sublineages and clinical characteristics in hospitalized patients in the southernmost state of Brazil
Source: BMC Infect Dis. 2024 Feb 13;24:193. doi: 10.1186/s12879-024-09089-3 (PMC10863127; doi:10.1186/s12879-024-09089-3)
Supplement: Supplementary file 1 — Supplementary Material 1. [file 12879_2024_9089_MOESM1_ESM.pdf]

Table Supplementary Material 1:

GISAID Identifier: EPI_SET_240202vg

doi: **10.55876/gis8.240202vg**

All genome sequences and associated metadata in this dataset are published in GISAID's EpiCoV database. To view the contributors of each individual sequence with details such as accession number, Virus name, Collection date, Originating Lab and Submitting Lab and the list of Authors, visit https://doi.org/10.55876/gis8.240202vg

Accession ID	Originating Laboratory	Submitting Laboratory	Authors
EPI_ISL_17238976, EPI_ISL_17238975, EPI_ISL_17238977, EPI_ISL_17238978, EPI_ISL_17238979, EPI_ISL_17238980, EPI_ISL_17238981, EPI_ISL_17238982, EPI_ISL_17238983, EPI_ISL_17238984, EPI_ISL_17238985, EPI_ISL_17238986, EPI_ISL_17394927, EPI_ISL_17238987, EPI_ISL_17238988, EPI_ISL_17238989, EPI_ISL_17238990, EPI_ISL_17238991, EPI_ISL_17238992, EPI_ISL_17238993, EPI_ISL_17238994, EPI_ISL_17238995, EPI_ISL_17394928, EPI_ISL_17238996, EPI_ISL_17238997, EPI_ISL_17238998, EPI_ISL_17238999, EPI_ISL_17239001, EPI_ISL_17239000, EPI_ISL_17239002, EPI_ISL_17239003, EPI_ISL_17239004, EPI_ISL_17239005, EPI_ISL_17321597, EPI_ISL_17239006, EPI_ISL_17239007, EPI_ISL_17239008	Laboratório de Microbiologia Molecular - Universidade Feevale	Molecular Microbiology Laboratory	Alana Witt Hansen, Fernando Rosado Spilki, Juliana Schons Gularte, Juliane Deise Fleck, Mariana Soares da Silva, Meriane Demoliner, Matheus Nunes Weber, Vyctoria Malayhka, Paula Rodrigues de Almeida, Micheli Filippi, Viviane Girardi
