# Supplementary material for: Diversity of Omicron sublineages and clinical characteristics in hospitalized patients in the southernmost state of Brazil
Source: BMC Infect Dis. 2024 Feb 13;24:193. doi: 10.1186/s12879-024-09089-3 (PMC10863127; doi:10.1186/s12879-024-09089-3)
Supplement: Supplementary file 2 — Supplementary Material 2. [file 12879_2024_9089_MOESM2_ESM.pdf]

Table Supplementary Material 2: Data of the sequences generated from the analyses by the CZ ID program.

Sample ID	Coverage Depth	Coverage Breadth	%id	GC Content	Total Reads	Mapped Reads
72451	291.3x	98.7%	99.8	37.9	86612	78968
72458	698.5x	98.8%	99.7	37.9	199582	183249
72480	363.7x	98.9%	99.8	37.9	103418	94996
72483	532.1x	98.7%	99.8	37.9	156572	142977
72488	399.9x	99.0%	99.8	37.8	112142	103790
72490	235.4x	93.8%	99.8	37.8	76380	65805
72494	583.8x	99.4%	99.8	37.8	165648	152663
72499	471.3x	98.8%	99.8	37.9	131944	122000
72509	514.3x	99.2%	99.8	37.8	145594	134429
72519	363.2x	98.6%	99.8	37.9	108278	98690
72533	249.2x	96.7%	99.8	37.9	73698	67052
72536	466.5x	64.5%	99.7	37.7	137056	125630
72544	449.8x	99.1%	99.8	37.9	135546	119809
72546	458.8x	99.3%	99.8	37.8	131280	120651
72554	154.6x	95.3%	99.8	37.8	46334	41337
72555	269.4x	98.7%	99.8	37.8	79654	72523
72559	600.9x	99.6%	99.8	37.8	178128	161887
72567	738.6x	98.8%	99.8	37.9	210928	194880
72574	367.4x	98.9%	99.8	37.9	102160	94669
72577	500.6x	98.9%	99.8	37.8	141312	130508
72579	343.2x	99.3%	99.8	37.9	100182	91360
72583	332.9x	98.8%	99.8	37.9	99416	90409
72584	551.2x	98.9%	99.7	37.8	156242	144134
72588	137.8x	98.6%	99.8	37.8	39154	35896
72591	180.8x	95.6%	99.8	37.7	100318	48238
72599	290.7x	97.5%	99.7	37.8	80728	74848
72602	415.2x	98.8%	99.8	37.8	119750	109306
72603	371.6x	98.2%	99.8	37.8	110192	100724
72604	566.3x	99.5%	99.7	37.8	162740	148908
72624	422.7x	99.4%	99.7	37.9	125194	113945
72655	492.8x	99.1%	99.7	37.8	144508	131969
72657	228.7x	94.7%	99.8	37.7	66148	60024
72667	546.5x	97.6%	99.8	37.8	154890	142785
72693	194.2x	88.8%	99.8	37.9	56818	51644
72698	229.9x	94.8%	99.8	37.9	69074	62639
72710	368.4x	86.5%	99.8	37.8	108554	98932
72712	194.9x	94.2%	99.8	37.8	58598	52225

Coverage Depth: average read depth of aligned contigs and reads over the length of the accession; Coverage breadth: percentage of the accession that is covered by at least one read and contig; %id: percentage of nucleotides of the consensus genome that are identical to those in the reference accession; GC Content: percentage of bases that are either guanine (G) or cytosine (C); Mapped Reads: Number of reads aligning to the reference accession.
